# Engineering Lipid Nanoparticles Beyond ApoE-Mediated Liver Tropism: Design Strategies for Extrahepatic and Organ-Selective Delivery

**DOI:** 10.3390/ijms27219818

**Published:** 2026-11-01

**Authors:** Hari Krishnareddy Rachamala, Gianluca Medigovic, Sandeep Atla, Ramcharan Singh Angom

**Affiliations:** 1 Department of Biochemistry and Molecular Biology, Mayo Clinic College of Medicine and Sciences, Mayo Clinic, Jacksonville, FL 32224, USA; angom.ramcharan@mayo.edu; 2 College of Allopathic Medicine, Nova Southeastern University, Fort Lauderdale, FL 33314, USA; gm1910@mynsu.nova.edu; 3 Texas A&M Drug Discovery Center, Department of Chemistry, College of Arts and Sciences, Texas A&M University, College Station, TX 77843, USA; sandeep.atla@tamu.edu

**Keywords:** lipid nanoparticles, apolipoprotein E, liver tropism, extrahepatic delivery, protein corona, organ-selective delivery

## Abstract

Lipid nanoparticles (LNPs) have evolved into a significant delivery technology for nucleic acid therapies, including messenger RNA (mRNA), small interfering RNA (siRNA), and genome editing tools. Although LNPs have demonstrated therapeutic success, they display strong tropism to the liver when systemically delivered, thus restricting their use for targeted delivery to extrahepatic organs. The major factor responsible for the liver targeting of LNPs is the binding of apolipoprotein E (ApoE) to the nanoparticle surface, which enables their recognition by lipoprotein receptors and uptake by liver cells. However, increasing evidence suggests that the tropism of LNPs can be altered by controlling lipid composition, physical and chemical properties, and acquired biological properties. This review discusses the mechanisms responsible for the liver tropism of LNPs as well as recent trends in modulating LNP tropism to extrahepatic organs. How ionizable lipids, helper lipids and sterols, PEG-lipids, LNP properties, and interaction with the protein corona can impact the organ tropism of LNPs as well as approaches for active and endogenous targeting of LNPs for organ- and cell-specific delivery are discussed. The challenges associated with organ- and cell-selective delivery of nucleic acids, such as delivery to functionally specific cell populations, endosomal release, species-specific translation, safety, repeated dosing, and clinical translation, are also described.

## 1. Introduction

Nucleic acid therapeutics have opened up new possibilities for manipulating biological functions, which is not easily achieved through existing small-molecule drugs and protein drugs. mRNA offers protein production in vivo, siRNA offers gene silencing in a sequence-specific manner, and genome editing is promising in altering disease-causing genes. However, the action of these nucleic acid molecules relies completely on delivery [1]. Nucleic acids are easily degradable, have low membrane permeability, and need to be transported to intracellular destinations before producing the desired biological effect. Therefore, progress in developing these nucleic-acid-based drugs has been accompanied by the development of nucleic acid delivery systems [2].

Lipid nanoparticles (LNPs) have gained importance in recent years as a potential non-viral carrier for this purpose due to their ability to deliver nucleic acids effectively, along with the flexibility of their physical and biological characteristics. Clinical use of LNPs proves the possibility of using synthetic lipids as a delivery system for nucleic acids on a large scale. But efficient encapsulation and cellular uptake are just two aspects of the entire process of delivery [3]. LNPs, after being administered systematically, have to navigate through various extra-cellular and intra-cellular barriers that include interaction with circulating molecules, biodistribution, recognition by certain cells, uptake by the cell, endosomal traffic, and release into cytosol.

One prominent limitation is the presence of considerable liver targeting by many systemically injected LNPs. The phenomenon not only results from the inherent physicochemical nature of the nanoparticle but is also influenced by the biological identity that it acquires when exposed to blood. Protein adsorption, especially by apolipoprotein E (ApoE), helps with interaction with lipoprotein receptors and liver uptake [4]. While this natural targeting phenomenon has been beneficial for therapies against liver diseases, it limits the use of these delivery systems in targeting extrahepatic organs and specific cells [5]. A growing amount of scientific literature shows that this biodistribution phenomenon cannot be viewed as an inherent feature of LNP technology. In fact, lipid composition, ionization behavior, surface chemistry, nanoparticle architecture, protein corona formation, targeting ligands, and mode of administration can all be utilized for modulating tissue and cellular tropism [6].

This knowledge brings a new perspective on the main issue in LNP development—not only should one estimate how effective the delivery of the nucleic acid cargo by the nanoparticle will be but also where and by what biological mechanisms this process takes place. This review is devoted to studying the molecular basis of ApoE-dependent liver targeting by LNPs and the design strategies for extending the capabilities of LNPs from the delivery of the nanoparticles to the liver predominantly. Special attention will be paid to the connection between LNPs’ physicochemical identity, protein corona formation, receptor recognition, biodistribution to organs, cell-specific internalization, intracellular trafficking, and functional nucleic acid activity.

### 1.1. LNPs for Nucleic Acid Delivery

Modularity of LNPs represents another reason behind the versatility of this platform. In general terms, conventional nucleic-acid-loaded LNPs include four main ingredients: ionizable lipid, helper phospholipid, cholesterol, and PEG lipid. All four ingredients play independent but complementary roles in the process of formation of the nanoparticles, as well as their stability and function [7].

Importantly, the pH-dependent characteristics of ionizable lipids are especially significant for the design of LNPs. Namely, at physiological pH, these lipids are electrically neutral and hence represent much less risk of interaction with cellular membranes compared to permanently cationic counterparts. After the uptake by cells and subsequent acidification of endosomes, increased protonation of the ionizable lipid leads to the formation of complexes with anionic lipids in the membrane that results in destabilization and the formation of non-bilayer structures, such as inverted hexagonal phases [7]. As a result, LNPs provide the means to overcome several barriers to nucleic acid therapies by protecting the cargo, enabling cellular uptake and intracellular release.

In addition, the modularity of the LNP platform allows for delivering different types of nucleic acid cargo. Today, the nucleic acids delivered using LNPs include plasmid DNA, mRNA, and siRNA, as well as some components of the CRISPR genome editing approach, such as Cas-encoding mRNA, guide RNA, and ribonucleoproteins [8]. Furthermore, lipid chemistry can be tuned to improve performance in the intracellular environment. In particular, multi-tailed ionizable phospholipids (iPhos) undergo pH-dependent structural changes leading to membrane destabilization and enable functional mRNA delivery and genome editing in vivo using the CRISPR–Cas approach [9]. Thus, it can be concluded that LNPs are more than passive vehicles for nucleic acids; their structure determines not only assembly but also membrane interaction and intracellular delivery [9].

Moreover, the possibility of altering the lipid chemistry provides the means to control the biodistribution of particles. In other words, the same features of the particle responsible for encapsulation and intracellular release can be used to influence the particle’s properties, which determine its interaction with the circulation system and subsequent tissue targeting. Therefore, understanding the performance of LNPs requires analysis of both intracellular delivery and distribution of the particle after administration.

### 1.2. The Challenge of Liver-Dominant Biodistribution

After intravenous injection, many standard lipid nanoparticle (LNP) formulations tend to accumulate in the liver due to its particular tropism. Hepatic tropism of the LNPs depends on the combination of many physiological characteristics of the liver, including fenestrated sinusoidal vessels, as well as the activity of resident hepatocytes, liver sinusoidal endothelial cells, and Kupffer cells able to interact with the LNPs [4,10]. However, liver targeting ability is strongly affected by changes of the particle surface after entering the bloodstream.

Many biological molecules quickly adsorb on the nanoparticle’s surface after intravenous injection, creating the so-called protein corona, which affects recognition by different cell receptors. ApoE is significant because its adsorption on the surface of the LNP will increase recognition by lipoprotein receptors, including LDLR, and thus will facilitate their hepatic uptake [4,5,11]. Therefore, in vivo interacting LNPs differ from the original particles due to the formation of the biological layer composed of adsorbed biomolecules.

In practice, hepatic tropism is not an inevitable feature of LNPs, as it can be changed by changing certain physicochemical characteristics that regulate the interaction with the biological environment. For instance, selective organ targeting (SORT) allows for achieving targeted delivery into extrahepatic organs such as the lungs or spleen. Addition of another lipid component to the standard formulation of LNPs can affect their surface charge and apparent pKa, as well as the interaction with circulating biomolecules, resulting in redirection of nucleic acids from the liver to other organs [12]. Thus, the biodistribution of the LNP is affected by interactions established in the organism and thus can be controlled.

The protein corona acts as a very important interface between the engineering features of the LNP and its subsequent biological interactions. Certain alterations in the composition of the lipids as well as the surface properties can change the acquired biological identity and, consequently, affect receptor recognition and organ targeting [12]. Details of the specific protein–receptor mechanism of ApoE-dependent hepatic uptake and the targeting of other organs are described in Section 2.

### 1.3. Need for Extrahepatic and Organ-Selective Delivery

Regulation of this pattern is crucial for development of LNP-based therapies that extend beyond applications limited to the liver. There are a number of possible applications that demand delivery of functional nucleic acids to cells within the lungs, spleen and immune system, tumor, brain and central nervous system, pancreas, muscle, and other non-liver organs. It has been shown that this redistribution can be achieved through various modifications to lipid composition, surface charge, targeting ligands, particle characteristics, or route of administration [13,14].

Notably, successful delivery of LNP to non-liver organs does not only require a decrease in hepatic targeting. The LNP should be delivered to the target organ, access the target cellular population, undergo productive internalization, escape the endosomal pathway, and deliver enough cargo in order to achieve the desired effect. Therefore, organ and functional delivery are two different endpoints of the LNP delivery process. It becomes particularly important for potent cargos like genome-editing systems, where undesired expression in additional cellular populations may lead to long-term adverse off-target effects [13,15]. Increases in tissue and cellular selectivity may potentially improve the therapeutic index of such therapy due to the concentration of the effect at the target location and decreased systemic distribution. These new requirements have led to the development of more programmable LNP delivery platforms. There are currently several methods that aim to change endogenous protein recruitment, use tissue-specific receptor pathways, include active targeting ligands, and combine organ-specific targeting with intracellular selectivity [15,16,17].

Thus, this review considers LNP biodistribution as a multiscale process connecting the molecular characteristics of lipids, the physicochemical properties of nanoparticles, acquired biological identity, receptor binding, organ distribution, cellular uptake, intracellular trafficking, and, finally, functional nucleic acid activity. We discuss the mechanism of ApoE-induced hepatic targeting and engineering approaches that could change this default pattern using ionizable lipids, helper lipids, sterols, PEG lipids, surface properties, and protein corona interactions. Next, we consider organ and cell-selective targeting, active and endogenous targeting of LNPs, and the challenges remaining in the context of endosomal escape, translation, repeated delivery, safety, and LNP discovery.

The current review builds on previous reviews on liver tropism and non-hepatocyte delivery of LNP formulations by emphasizing the transition in the mechanistic understanding from ApoE-dependent recognition in the liver to other recognition pathways that allow for selective targeting of organ/cell types [4,13,18,19,20,21]. The review addresses LNP design and physicochemical properties, changes in the protein corona structure, receptor recognition, nucleic acid function delivery, and translational issues in an integrated way towards engineering of LNPs beyond liver-specific delivery. Mechanistic insights are especially pertinent to the translational aspects of this issue, as effective delivery of LNPs beyond the liver requires not only successful delivery of LNPs out of the liver but also their reliable delivery into the target cell population, efficient delivery of cargo into cells, safety, manufacturability, and efficacy.

## 2. Why LNPs Target the Liver: The Role of ApoE

**Figure 1:**
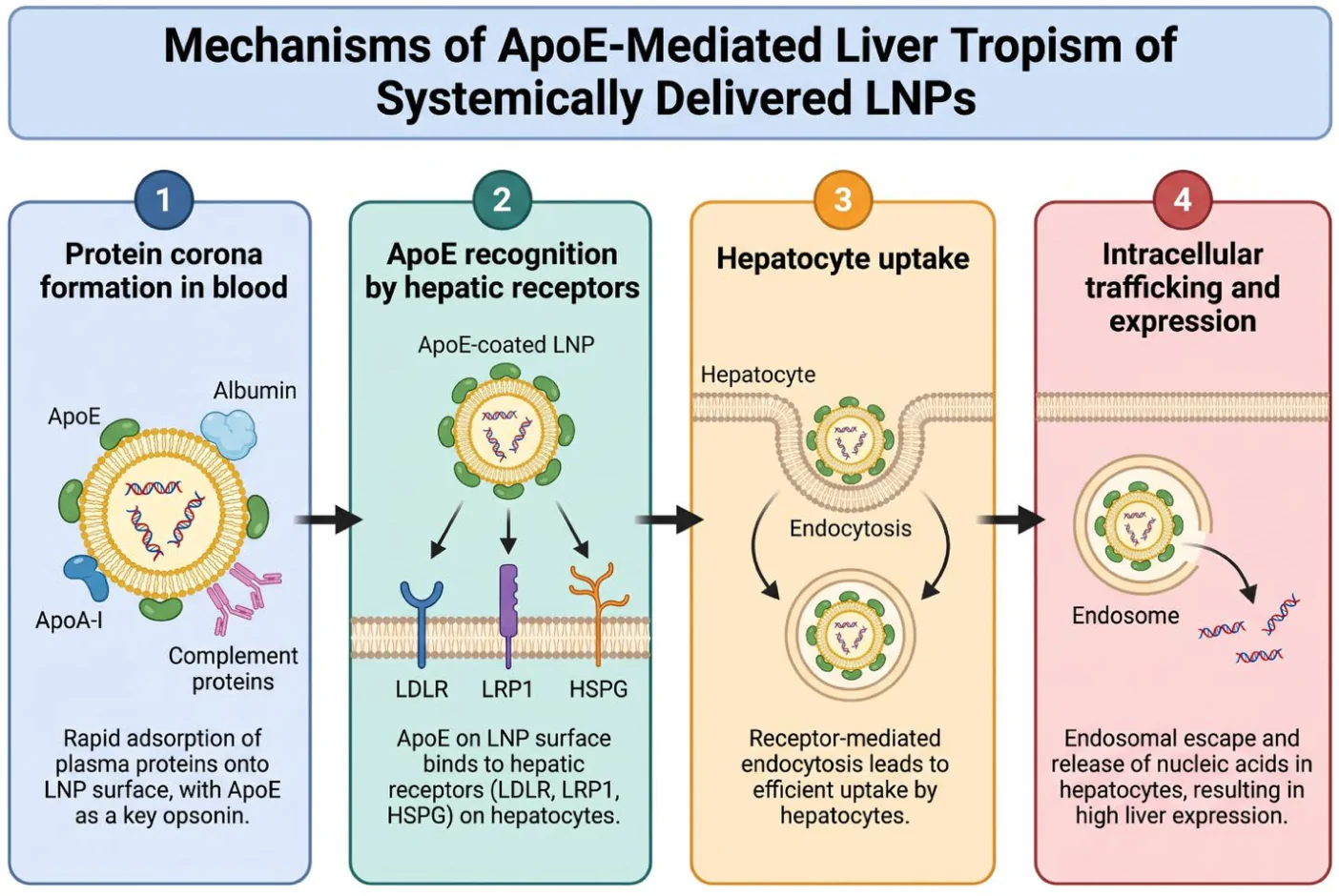
Process by which ApoE induces liver tropism of LNPs after systemic injection. During systemic injection, the LNPs are coated by the adsorption of plasma proteins to form the protein corona that includes ApoE. The ApoE-LNPs then interact with receptors present in the liver cells, making it easier for the LNPs to enter into the cell via receptor-mediated endocytosis. Abbreviations: ApoE, apolipoprotein E; HSPG, heparan sulfate proteoglycan; LDLR, low-density lipoprotein receptor; LNP, lipid nanoparticle; LRP1, low-density lipoprotein receptor-related protein 1. Created in BioRender. Rachamala, H. K. (2026) https://BioRender.com/3oq5l3j (accessed on 25 October 2026).

Due to interaction between specific physicochemical properties of LNPs and biological properties acquired in the course of their transition into the bloodstream, systemic administration of LNPs results in their selective accumulation in the liver. One of the biological mechanisms behind this phenomenon is the formation of plasma protein corona and the following association with ApoE. ApoE molecules adsorbed on the surface of LNPs serve as an endogenous targeting ligand and promote interaction with lipoprotein receptors and their liver-specific uptake. Recent studies have shown, however, that this mechanism is strongly dependent on LNP composition and is not universal or obligatory for all LNPs. Hence, knowledge about the role of nanoparticle characteristics in protein corona formation, ApoE interaction, and receptor engagement is critical for rationally designing LNPs with liver-targeting capabilities (Figure 1).

### 2.1. Protein Corona Formation

During the interaction with the biological fluid after systemic administration, nanoparticles form a complex of proteins adsorbed on their surface, namely, the protein corona. This biologically acquired interface alters the initial physicochemical properties of the nanoparticles, providing them with a novel biological identity recognized by the biological environment. Importantly, the protein corona does not completely cancel out the initial physicochemical properties of nanoparticles. It has been demonstrated that depending on the surface charge, the proteins were adsorbed on positively and negatively charged nanoparticles, while surface charge remained a key factor in affecting cellular uptake, lysosomal localization, and cytotoxicity [22].

Surface properties of nanoparticles considerably affect the composition of the protein corona. As mentioned above, PEGylation of the nanoparticle surface is one of the key ways to change nanoparticle surface characteristics. For instance, studies involving PEG–PLGA nanoparticles have revealed that increasing PEG surface density higher than approximately 20 PEG chains per 100 nm^2^ reduced ApoE content in the corona and prolonged systemic circulation, suggesting that PEG density regulated both corona composition and pharmacokinetics. Moreover, ApoE and clusterin act as dysopsonins depending on the surface characteristics of nanoparticles, while removal of these nanoparticles from circulation was associated with LDLR-mediated uptake [23]. Hence, apart from acting as a passive layer of adsorbed proteins, the protein corona actively determines the clearance of nanoparticles. Although there is a difference between PEG-PLGA nanoparticles and LNPs loaded with nucleic acids, the study shows that the level of PEG at the particle surface influences corona formation and ApoE binding, which is significant when designing nucleic-acid-loaded LNPs.

Formation of the protein corona is affected by environmental and particle-specific factors. The local pH and particle size may alter the strength of intermolecular interactions, which, in turn, affect protein adsorption. In the case of solid lipid nanoparticles, the transition from physiological to acidic pH changed the protein–particle interaction and increased size-dependent aggregation, while small hydrophilic particles were more resistant to aggregation [24]. Hydrophobicity of the particle surface is another critical property regulating the formation of the protein corona. Nanoparticles arranged along the controlled hydrophobicity gradient showed that hydrophobic surfaces adsorbed approximately 2.1-fold higher amounts of serum proteins compared to hydrophilic ones, while hydrophilic nanoparticles had a higher rate of exchange between the surface-bound and soluble proteins [25].

All of these relationships are especially important for LNP delivery, as the proteins present on the nanoparticle surface determine the receptors that will recognize the nanoparticle. Thus, the formation of the protein corona acts as a mechanistic link between LNP surface properties and receptor engagement. Among numerous proteins interacting with lipid-based nanoparticles, ApoE plays a key role in determining the recognition of LNPs by the liver.

### 2.2. ApoE Adsorption onto LNPs

ApoE is an endogenous apolipoprotein involved in physiological lipid transport and can become a constituent of the protein corona formed by systemically delivered LNPs. Being bound to the surface of LNPs, ApoE becomes an endogenous ligand capable of interacting with lipoprotein receptors expressed by liver cells. The amount of ApoE recruitment and consequent dependency on ApoE for LNP delivery are strongly dependent on the lipid composition of the LNPs.

It was shown that high-throughput screening of ionizable LNPs differing in structure can prove the dependence of ApoE binding. Testing of 60 LNP formulations in wild type, ApoE−/−, and LDLR−/− mice revealed that rather small changes in hydrophobic structure of ionizable lipids can significantly change ApoE dependency. Lengthening the hydrophobic tail of diketopiperazine-based ionizable lipids from cKK-E12 to cKK-E15 decreased dependence on endogenous ApoE and LDLR and still retained efficient cytosolic delivery and function of mRNA and corresponding protein expression [26]. Hence, ApoE binding is not a universal property of ionizable LNPs but rather a tunable property that depends on lipid composition.

Surface modification is another tool used to control protein recruitment. Electrostatic deposition of carboxylated polyanions, such as hyaluronic acid and poly(amino acid), conjugates onto the surface of positively charged LNPs, which produces layered LNPs that retain nucleic acid encapsulation and achieve a fourfold reduction in total protein adsorption. Surface modification altered the apparent pKa, cellular uptake, intracellular trafficking, and organ biodistribution [27]. This shows that manipulation of the electrostatic interaction between LNP and biological surroundings allows one to inhibit non-specific protein recruitment and perhaps ApoE-dependent hepatic recognition.

PEGylation also affects ApoE’s ability to interact with the surface of LNPs. Incorporation of 5 mol% of DMG-PEG2000 in the DC-Chol nanoparticles provided a steric barrier, which decreases ApoE binding strength, reduces direct protein-lipid contact, and causes ApoE structure perturbation. It also causes delayed accumulation of nanoparticles in the liver [28]. Consequently, PEG lipid content and accessibility affect not only particle stability but also biologically important interactions, which define organ distribution.

Nanoparticle morphology is an additional parameter that can be used for the control of ApoE recruitment. Partial replacement of conventional cholesterol with 3β-modified TLR7/8 agonist-conjugated cholesterol resulted in rigid flower-like LNPs with changed surface curvature. Under physiological flow conditions, this led to 40% decreased ApoE adsorption and a decrease in ApoE-dependent hepatic tropism and hepatotoxicity [29]. Overall, these studies show that ionizable lipid structure, surface charge, PEG shielding, sterol composition, and nanoparticle morphology affect ApoE recruitment. Thus, ApoE adsorption is an engineerable property that arises from LNP composition and not an inherent one due to systemic administration.

### 2.3. ApoE–LDLR-Mediated Hepatic Uptake

Upon binding of ApoE to the surface of the lipid nanoparticle (LNP), the ApoE moiety is capable of functioning as an endogenous targeting ligand for receptors of the lipoprotein receptor class, specifically the low-density lipoprotein receptor (LDLR). In this way, there is a mechanistic link established between the process of protein corona formation in circulation and subsequent uptake. The physiological ApoE–LDLR pathway plays an important role in regulation of lipid metabolism in liver, while experimental interference with any of its components leads to drastic alteration of receptor-mediated lipid metabolism and hepatic clearance of lipids [30]. Thus, ApoE-capturing LNPs utilize the natural physiological transport pathway instead of employing some exogenous targeting ligand.

Manipulation of ApoE-mediated receptor binding also demonstrates the significance of this pathway for biodistribution of LNPs. Experimental competitive inhibition of ApoE-mediated receptor recognition via the use of NanoPilot™ platform resulted in three-fold decrease of off-target hepatic mRNA expression and clearance, along with forty-fold increase in targeted T-cell transfection both in vitro and in vivo. Thus, interfering with ApoE-mediated receptor recognition reduced sequestration in the liver and promoted the delivery of the LNP payload to the extrahepatic target cell type [31].

As an alternative approach, the nanoparticles themselves may be engineered to avoid the binding of ApoE prior to their receptor recognition. In this respect, design of ionizable cholesteryl lipids, which combined the properties of both ionizable lipid and cholesterol components within one molecule, resulted in the formation of stable three-component LNPs that exhibited significantly altered protein corona formation. These LNPs showed a 90% reduction in ApoE binding compared with regular four-component LNPs along with reduced ApoE/LDLR-mediated endocytosis of the nanoparticles in hepatocytes and decreased hepatic mRNA delivery [32]. Thus, the connection was established between the molecular structure of the lipid, ApoE binding, receptor-mediated cellular uptake, and biodistribution.

Overall, the data described above indicate the mechanistic sequence through which LNP composition impacts ApoE binding, ApoE-containing protein corona influences receptor recognition, and receptor recognition facilitates hepatic uptake. However, this sequence must not be perceived as the only mechanism of action responsible for LNP delivery into the liver and elsewhere. The dependence on ApoE and LDLR is very different between various formulations, thus implying the participation of other protein corona and receptor pathways in the biodistribution of LNPs.

### 2.4. ApoE-Dependent and ApoE-Independent Pathways

While the role of receptor recognition by ApoE in the hepatic specificity of many lipid nanoparticle (LNP) formulations offers a plausible explanation for the phenomenon, ApoE is far from being an obligatory factor in LNPs’ biodistribution profile. Dependence on ApoE is heavily regulated by the chemical composition of nanoparticles and the biological identity formed through interaction with blood plasma components. The significance of this fact is especially high in extrahepatic delivery scenarios, as changes in lipid composition can shift LNP trafficking from an ApoE-mediated process to alternative biological targeting [4,6].

The direct evidence of formulation-dependent behavior came in the course of studies of Selective Organ Targeting (SORT) LNPs. Depletion of ApoE drastically impaired the functional delivery of both regular and liver-targeting LNPs. On the contrary, lung-targeting SORT LNPs preserved their efficiency in ApoE−/− mice, while spleen-targeting SORT LNPs demonstrated increased splenic delivery in the absence of ApoE [5]. These results clearly show that extrahepatic LNP delivery can occur independently of ApoE and that reduction of an ApoE-mediated pathway allows alternative organ-specific targeting mechanisms to become functional.

Notably, this formulation-dependent behavior corresponds to different protein coronae compositions. While liver-directed conventional delivery occurs via ApoE-dominated targeting, spleen-targeting SORT LNPs demonstrate a preference towards β2-glycoprotein I, and lung-targeting SORT LNPs have vitronectin enriched protein coronae [5]. Hence, a change in LNP composition does not result in the elimination of protein-corona-mediated targeting but rather replacement of ApoE-mediated biological identity by another protein corona capable of engaging other pathways and changing organ specificity.

Thus, the ApoE–LDLR model of LNP biodistribution must be expanded to consider the role of protein corona formation. Different LNP compositions can lead to the formation of unique biological identities, thus indicating that extrahepatic targeting can occur not due to blocking ApoE absorption but changing the balance of plasma proteins engaged in the process of binding to endogenous receptors.

## 3. Engineering LNPs to Overcome Liver Tropism

**Figure 2:**
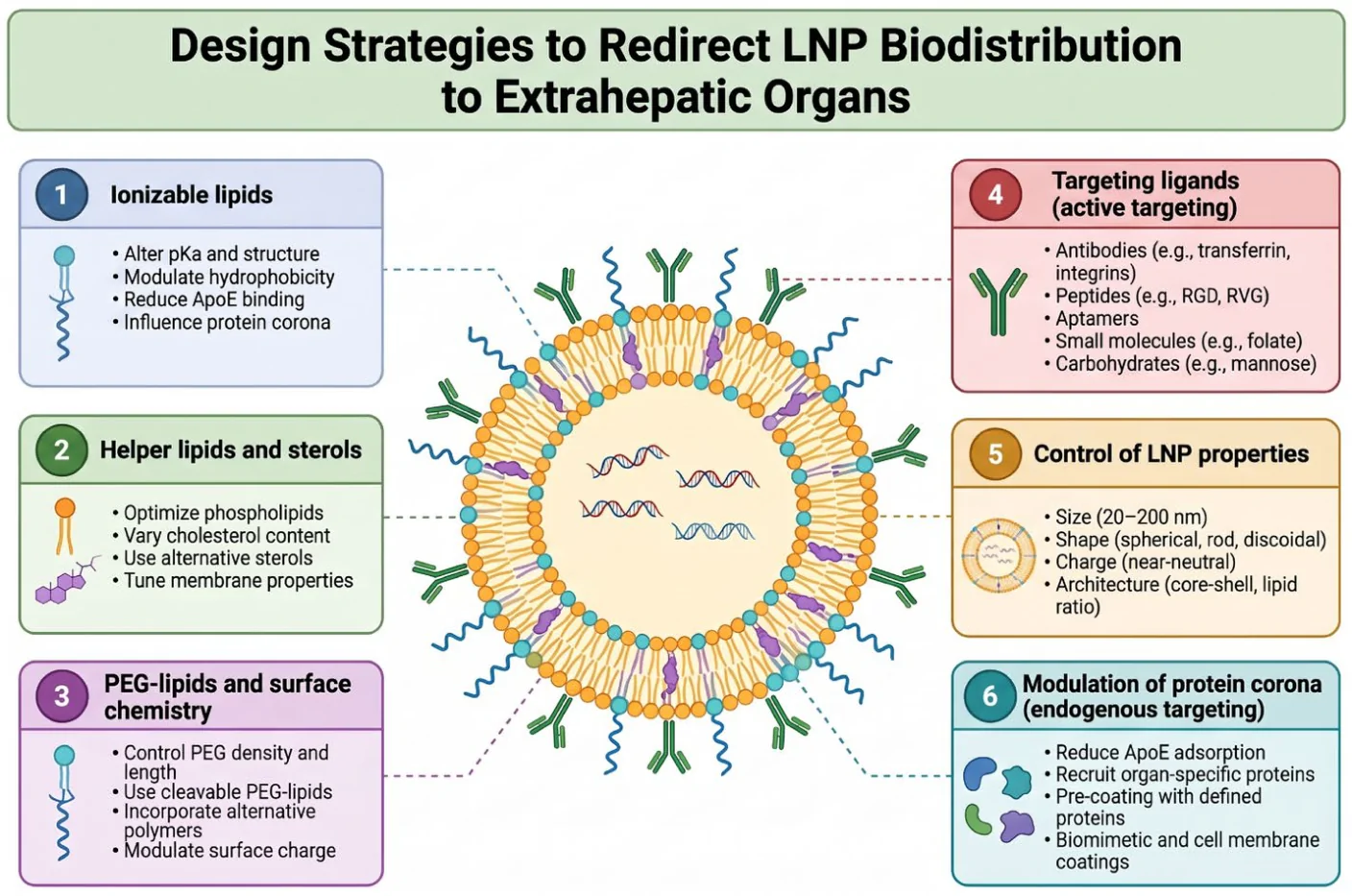
Strategies for tailoring LNPs for redistribution from liver-centric to extrahepatic tissue biodistribution. LNP selectivity could be fine-tuned using ionizable lipids, helper lipids and sterols, PEGylated lipids and surface modifications, targeting ligands, physical–chemical characteristics, and protein corona. All of these strategies could help overcome ApoE-mediated targeting to the liver and achieve organ-specific as well as cellular distribution outside of the liver. Abbreviations: ApoE, apolipoprotein E; LNP, lipid nanoparticle; PEG, polyethylene glycol; pKa, acid dissociation constant. Created in BioRender. Rachamala, H. K. (2026) https://BioRender.com/izz6op8 (accessed on 25 October 2026).

ApoE targeting is formulation-dependent, thus offering a key route towards controlling the biodistribution of LNPs. Rather than being restricted to their natural propensity for the liver, LNPs gain organ- and cell-specific tropisms via the effects of lipid chemical nature, ionization profile, surface properties, structural architecture, and the biological environment surrounding the particles [6,33]. Consequently, management of liver-specific targeting requires the manipulation of nanoparticles’ physical and chemical properties as well as the biological identity it acquires once delivered into the body. Important parameters for manipulation include ionizable lipid chemistry and pKa value, helper lipids and cholesterol, PEG lipid chemistry, surface charge and morphology, ligands for targeting, and interaction with the corona, as shown in Figure 2.

### 3.1. Ionizable Lipid Chemistry and pKa

The ionizable lipid is one of the key determinants of LNP efficacy, as its protonation controls nucleic acid encapsulation, LNP surface charge, membrane interaction, and endosomal escape [34]. The most common design of ionizable LNPs maintains their neutrality at physiological pH and protonates them within acidic conditions inside of endosomes. The charge switch reduces nonspecific electrostatic interactions during circulation and, after cell internalization, increases interaction with anionic endosomal lipids [7,34].

Modification of the ionizable lipid headgroup may dramatically affect apparent pKa and LNP tissue delivery. Ionizable lipids with an oxabispidine backbone, which contains a rigid bicyclic diamine headgroup, resulted in LNPs with an apparent pKa of 8.82 that was significantly higher than the one for the benchmark MC3 LNPs (pKa of 6.40). After intratracheal administration, the optimized ionizable lipid LNP showed a 43-fold increase in eGFP expression in rat lungs in comparison with MC3 LNPs and transfected pulmonary macrophages and epithelial cells without neutrophil recruitment [35]. While the administration route plays a significant role in lung localization, this example demonstrates how the modification of the headgroup allows for altering the ionization properties and functional delivery of LNPs.

For many systemic delivery LNPs, the optimal apparent pKa value may be close to 6–7, which provides a good balance between near-neutral LNP circulation and LNP protonation after endosomal acidification. SM-102 ionizable lipids LNPs, with an apparent pKa of 6.3–6.5, demonstrate the transition from a strong positive charge under acidic conditions to near-neutral charge at physiological pH with highly efficient nucleic acid encapsulation [36]. Importantly, the addition of surface aptamer on LNPs did not significantly change this pKa, indicating that active targeting can be introduced to LNPs along with maintaining the pKa necessary for intracellular delivery [36].

However, apparent pK value does not determine biological behavior. 5A2-SC8 and 3A5-SC14 ionizable lipid LNPs had a close size but identical apparent pKa of 6.5 and still showed a difference in cellular tropism in the liver. In particular, 5A2-SC8 LNPs with an ApoE-enriched protein corona showed approximately 87% silencing of factor VII in hepatocytes, while 3A5-SC14 LNPs were enriched in albumin, localized mainly in Kupffer cells, and did not cause efficient silencing of hepatocytes [37]. Therefore, relatively small differences in ionizable lipid structure enabled alteration of protein corona composition and cellular recognition despite identical physicochemical properties of LNPs.

In addition to the exemplary instances mentioned above, there have been broad investigations of ionizable lipids wherein the structural components of the lipid, namely the headgroup structure, linker chemistry, tail structure, and degradability, have been shown to significantly affect LNP potency and tissue selectivity [3,9,38,39]. Combinatorial screening of multi-tailed ionizable phospholipids (iPhos) indicated structure-based control of endosomal release and tissue-specific delivery, such as to the liver, spleen, and lungs [9]. Moreover, ionizable lipids containing piperazine moieties have shown structure-dependent mRNA delivery in specific immune cell types in the absence of ligand-mediated delivery [38]. Recently, directed chemical evolution of biodegradable ionizable lipids has resulted in the discovery of structure–activity relationships based on the headgroup, ester linker, and hydrophobic tail domains [3,39]. Machine-learning-based combinatorial screening of large chemical spaces is now being utilized in discovering potent ionizable lipids.

### 3.2. Helper Lipids and Cholesterol

Despite the significant research devoted to the ionizable lipids, helper lipids and cholesterol affect LNP construction, membrane organization, cargo retention, and interactions with cellular membranes in a very context-dependent way. Unlike ionizable lipid components, helper lipids and cholesterol are structurally engaged in intermolecular complexes and do not have simple passive roles in formulation.

Replacement of traditional phospholipid helper lipids by monoglyceride-type non-phospholipids illustrates this feature. When DOTAP-based nanoparticles were prepared using monoolein instead of DOPE along with optimization of cholesterol loading, better mRNA transfection and more pronounced in vivo mRNA expression in the lungs and spleen were observed [40]. This suggests that helper lipid composition is able to modify the membrane architecture and fusion capabilities of LNP sufficiently to affect its internalization and organ-specific delivery.

Helper lipids affect the LNP internal structure itself. It has been shown that polar helper lipids are involved in the formation of multilamellar structures related to encapsulated nucleic acids in KC2 containing siRNA LNPs. On the contrary, nonpolar triglycerides were much less effective in supporting cargo retention. Thus, the appropriate helper to ionizable lipid ratio had to be provided to keep nucleic-acid-loaded LNPs structured and loaded [41]. This implies that helper lipids are involved in the formation of the LNP structure rather than simply surrounding the formed ionizable lipid–cargo complex.

Cholesterol is able to affect LNP structure and functional properties, as well. In cationic siRNA lipoplexes, addition of cholesterol resulted in more efficient pulmonary accumulation after intravenous administration. But functional lung gene silencing in such lipoplexes depended on the degree of interaction between cholesterol and saturation of the hydrophobic chain of cationic lipid [42]. In other words, more pronounced organ accumulation does not necessarily mean better delivery of functional nucleic acid cargo.

More recent research suggests that even sterol chemistry could become an organ targeting variable. Introduction of ionizable sterols as another component of LNPs led to the generation of mRNA expression, which could select for either the spleen or lungs depending on sterol structure and amount [32,43].

### 3.3. PEG Lipid Composition

PEG lipids represent a relatively small molar fraction in LNPs, but they have a tremendous impact on the process of particle formation, colloidal stability, protein accessibility, cellular uptake, and biodistribution. The main characteristic of PEG is the hydration shell providing the steric layer and preventing particle aggregation and non-specific interaction. At the same time, excessive PEG shielding may affect the proper packing of the lipid bilayer and cellular interactions, demonstrating one of the numerous trade-offs existing between particle stability and cellular entry.

One such trade-off is clearly visible in the course of particle formation. The use of microfluidic mixing resulted in heterogeneous particles and the formation of micrometer-sized aggregates in the case of DSPE-PEG2000 at 5 mol% in rigid DSPC-based formulations, while decreasing the molar fraction of PEG lipids down to 2.5 mol% led to the formation of extremely monodisperse particles with approximately 100 nm diameter [44]. Therefore, PEG lipids might be responsible for the control of lipid assembly, rather than the modification of the particle surface characteristics.

PEG lipids provide the means for post-assembly particle surface functionalization. In the case of trastuzumab–PEG lipid insertion into preformed liposomes, the proper composition of PEG lipid and cholesterol contents was important to achieve effective antibody insertion and to preserve colloidal stability [45]. Thus, PEG lipids might provide steric stabilization and target molecule anchoring at the same time.

At the cellular level, the increase in PEG density leads to a decrease in the interactions between nanoparticles and cell membranes. MC3-based LNPs with higher PEG lipid concentrations had smaller particles and slow cellular uptake, resulting in different dynamics of PD-L1 silencing. Low PEG content resulted in fast and short activity, while high PEG content led to delayed uptake and prolonged functional silencing [46].

Moreover, PEG anchor chemistry, rather than its concentration, is responsible for the distribution pattern. The latest systematic study of mRNA-LNPs showed that PEG lipid tail length influences organ localization: formulations containing shorter C8 ceramide PEG had higher lymph node localization, while LNPs with longer PEG lipid anchors had more pronounced liver accumulation [47].

### 3.4. Surface Charge, Size, and Morphology

The emergent properties of liposomes used as nanoparticles depend on their chemical composition, as they are responsible for surface charge, size, and morphology. These three properties in turn govern interactions with plasma proteins, vascular barriers, the extracellular matrix, and cell membranes and serve as additional factors in the determination of nanoparticle biodistribution.

The role of the surface charge is especially significant, as electrostatic interactions influence both protein adsorption and binding to cells. Quantification of mRNA-LNP vaccines using imaged capillary isoelectric focusing has shown that the apparent surface isoelectric points ranged between approximately 7.7 and 8.1, implying that the surface charge was mainly determined by the concentration and ionization of the cationic lipids rather than by mRNA cargo [48]. In other words, the surface charge is an emergent property of the formulation chemistry that might be independent of the nucleic acid cargo.

Size and morphology affect biological interactions, as they determine vascular transportation and cell uptake mechanisms. Research into nanocarriers of various sizes, surface charges, and morphologies has demonstrated that these factors influence membrane binding and internalization [49]. Surface coatings that can adjust both the size and zeta potential might affect tissue residence via electrostatic interactions [50]. However, although these studies highlight the design principles of nanoparticles, the use of these principles in the case of nucleic acid LNPs requires considering dynamic changes in surface structure and the formation of the protein corona.

In particular, morphology influences LNP biodistribution indirectly through protein recruitment. As has been shown above, sterol-induced formation of flower-shaped particles decreases surface curvature and limits ApoE adsorption [29]. The size, charge, and shape of the particles must not be considered merely as physical characteristics but also contributors to the biological identity of LNPs through modulation of proteins interacting with the particle surface.

### 3.5. Modulating the Protein Corona

In consideration of the role of the formation of the protein corona in linking the physicochemical properties of LNP formulations with their biological recognition, the deliberate manipulation of this interfacial layer becomes a key approach to overcoming the inherent hepatotropic properties of these particles. As explained in Section 2.4, different LNP formulations can acquire different biological identities following exposure to circulating biomolecules. Therefore, the engineering problem consists of not only preventing protein adsorption but also controlling how formulation composition and physicochemical properties affect protein abundance, accessibility, and exchange at the LNP–biomolecule interface [51].

The following physicochemical factors play a role in this process. Surface charge affects the strength of electrostatic interactions with circulating biomolecules, while PEG density and PEG lipid anchoring chemistry control steric accessibility of the underlying lipid surface. Lipid hydrophobicity, sterol content, particle morphology, and surface functionalization can also change the affinity and organization of the adsorbed proteins. In this respect, even small changes in LNP composition can result in significant differences in the biological interface available for cells in spite of conventional parameters like particle size or apparent pKa remaining unchanged. Some empirical data from studies of other nanoparticle systems confirm the possibility of deliberate control over corona composition [51]. For example, changes in crosslinker chemistry in mannose-modified chitosan nanoparticles resulted in alteration of surface accessibility and increased the amount of apolipoprotein B48 in the protein corona, thus affecting macrophage uptake [52]. Manipulation of nanoparticle size, charge, surface ligands, and coatings and pre-adsorption of certain proteins can change the balance between opsonic and dysopsonic proteins and affect the immune recognition and biodistribution of nanoparticles [53]. Small molecules can also affect competitive adsorption of proteins; for example, phosphatidylcholine supplementation had a significant effect on the abundance and diversity of proteins in the nanoparticle coronas [54]. Though these nanoparticle systems are not conventional nucleic acid LNPs, they show that the composition of protein coronas depends on controllable physicochemical and environmental factors.

In the case of LNPs, this idea is particularly relevant as the formulation variables mentioned in the current section, namely, ionizable lipid chemistry, the composition of the auxiliary lipids and sterols, PEG lipid chemistry, surface charge, and particle morphology, come together at the nanoparticle–biological interface. The combination of these variables determines which regions of the particle are exposed to circulating biomolecules and affect its biological identity. Therefore, protein corona engineering should be viewed as an emerging property of formulation design, rather than an additional modification of the LNPs after their formation.

All of the above shows that the engineering of extrahepatic LNPs does not consist in optimizing any formulation variable. Ionizable lipid chemistry controls the processes of ionization and interaction with membranes; composition of the auxiliary lipids and sterols affects structural organization; PEG lipids affect surface accessibility; and particle charge, size, and morphology control the interactions with the surrounding biological environment. Overall, these factors determine the composition of the protein corona formed around the particle and receptor interactions. The combination of these variables gives an opportunity to design the LNPs with shifted target recognition from ApoE-dominated hepatic recognition towards programmed organ selectivity. The major engineering variables influencing LNP tropism and ApoE-dependent liver targeting are summarized in Table 1.

In conclusion, the above-discussed engineering strategies offer different, although complementary, approaches for LNP delivery into an organ beyond the liver. Firstly, engineering ionizable lipids provides an exceptionally wide design space and allows for manipulating multiple aspects of delivery, including nucleic acid loading, endosomal escape, and tissue targeting. However, ionizable lipids show complex SAR relationships that often require extensive screening. Secondly, engineering helper lipids and sterols constitutes a relatively simple way of modulating membrane physical parameters and biodistribution. Yet these effects can be very formulation dependent. Thirdly, engineering PEG lipids can provide enhanced stability and circulation half-life and manipulation of protein access; however, over-PEGylation may negatively impact uptake and delivery. Finally, changing the particle size, surface charge, or shape may affect biodistribution, which depends on all three factors that are, however, intertwined and can be adjusted by the interaction of the LNPs with biologically relevant liquids. Lastly, protein corona engineering offers an opportunity to use endogenous targeting pathways without introducing any additional ligands; however, the corona is dynamically modified depending on the formulation and the biological environment and can be different among species. Thus, no one technology is universally better, and the best approach probably lies in combining lipid chemistry, physicochemical characteristics, and biological targeting according to the desired organ, cell type, and route of administration.

## 4. Engineering LNPs for Extrahepatic Organ Targeting

The ability to regulate LNP composition, surface properties, and biological identity has led to the extension of nucleic acid delivery from the liver to various types of extrahepatic organs. Notably, organ targeting can be achieved through various means. Changes in lipid composition may influence surface charge and protein corona formation, thus redirecting receptor binding, whereas ligand-based targeting can promote specific cellular uptake via receptors. Moreover, the mode of administration may circumvent systemic limitations, and physical forces may be used as an external way to direct delivery. Together, these approaches have enabled functional delivery of LNPs to the lungs, spleen and immune system, tumors, CNS, heart, kidney, pancreas, and other organs that are difficult to target.

### 4.1. Lung Targeting Strategies

The lung is increasingly recognized as an easily accessible target tissue outside of the liver for the delivery of nucleic acids using LNP platforms. The extensive vasculature, the vast endothelial surface area, and the diverse collection of epithelial and immune cell populations allow for both systemic and local pulmonary delivery. Moreover, pulmonary tropism can be imparted to systemically administered LNPs through the modification of lipid composition to bypass the first-pass effect and liver uptake.

Specifically, the use of permanently cationic helper lipids was shown to enable selective organ targeting. The LNP formulation made up of permanently cationic helper lipid 18:0 DDAB along with cKK-E12, cholesterol, and C14PEG2000 showed selective transportation of mRNA to the lungs, delivering to more than 50% of pulmonary endothelial cells, as well as some parenchymal and immune cell populations [55]. Transcriptomic analyses of single-cell sequencing of LNP-transfected cells confirmed the induction of RNA processing, translation initiation, and ribosomal assembly pathways [55]. Therefore, delivery to this organ is possible not only through accumulation at the organ level but also by utilizing the intrinsic ability of target cells to process and translate delivered mRNA.

Furthermore, the ionizable lipid structure can increase specificity. Upon intravenous injection, the optimized sulfonium lipid LNPs showed 99% specificity towards the lung tissue, which is measured by the percentage of bioluminescence emitted from the major organs in the body that was specifically from the lung tissue [56]. The study provided important evidence that the organ-specific delivery can be successfully used to treat diseases.

In turn, the route of delivery rather than biodistribution can be altered to achieve pulmonary targeting. Solid LNP containing silibinin demonstrated prolonged retention and much higher lung exposure in comparison with the drug alone when administered directly into airways [57]. While this platform does not contain nucleic acids, the results obtained demonstrate the principle that local pulmonary delivery may result in decreased exposure of the rest of the body and the liver [57]. In summary, composition-driven organ targeting and the local pulmonary delivery represent complementary approaches.

### 4.2. Spleen and Immune Cell Targeting Strategies

The spleen is another vital non-hepatic site to target owing to its high density of B cells, T cells, dendritic cells, and macrophages participating in antigen presentation and systemic immune responses. Spleen-specific LNPs can thus find applications in vaccination, immunotherapy for cancer, and immune reprogramming.

First, modification of LNPs through metal coordination has been used in an attempt to improve both structure and biological properties. Zinc-coordinated LNPs using imidazole-based lipids carrying mRNAs encoding antigens enabled significant spleen-specific gene expression, as well as improved dendritic-cell-mediated antigen presentation and antigen-specific CD8+ T-cell response [58]. Using this combination, treatment of a melanoma mouse model resulted in tumor growth inhibition, thus demonstrating how the carrier molecule itself might participate in immune response elicitation through this therapy.

More specific targeting of splenic cells might be possible through the interactions of the carrier molecule with the host biology processes. Lipids with ionizable moiety TOT-5 and increased DSPC content allowed for obtaining LNPs with rigid and charge-neutral surfaces to avoid ApoE-mediated hepatocyte uptake and selectively deliver mRNA to the marginal zone B cells in the spleen [59]. The mechanism underlying this phenomenon involved deposition of the complement factor C3, enabling receptor-dependent CD21/35-mediated cell targeting. This example is particularly interesting, as it demonstrates how alteration of the helper lipid composition could allow for replacing one natural targeting pathway with another one without using exogenously added ligands.

Another approach uses biomimicry and involves fusion of LNPs with senescent red blood cells. Such fusion exploited the physiological process of erythrophagocytosis to deliver particles to the spleen and enabled their uptake by the macrophages and dendritic cells there. Delivery of rapamycin in such particles led to reprogramming of splenic immune cells towards anti-inflammatory phenotypes and improved symptoms in the mouse colitis model [60]. Thus, these examples demonstrate how splenic targeting could be achieved through metal coordination, complement recruitment, or incorporation of natural membranes. Even though this method involved the delivery of rapamycin instead of DNA, it has shown an approach that is easily replicable for use in delivering nucleic acids based on the idea of using components from nature to improve targeting of the spleen.

### 4.3. Tumor Targeting Strategies

On the other hand, tumor-directed lipid nanoparticle delivery is a much more difficult task because of the heterogeneous nature of solid tumors in terms of vascular permeability, extracellular matrix structure, presence of receptors, immune cell populations, and interstitial pressure. As a result, effective tumor delivery often implies not only bypassing the liver but also combining the physicochemical approach with active or endogenous biological recognition [61].

One possible way to deal with this problem is data-driven formulation screening. Machine learning techniques were used to search for the optimal formulation in the wide space of lipid compositions to develop PEG-free, negatively charged exosome-like nanoparticles made up of natural lipids. The developed formulations showed high cellular uptake in cervical, lung, and breast cancer cell lines while having good cell viability [62]. Though these findings demonstrate only tumor cell uptake and not tumor-specific biodistribution, they show the potential of computational methods in fast screening of complicated lipid compositions.

Surface functionalization is another approach that combines passive accumulation in the tumor with receptor-mediated uptake. Modified solid and hybrid LNPs with the surface decorated by chondroitin, lactoferrin, or galactose exhibited better tumor cell uptake and accumulation compared to non-targeted particles [63,64]. Nevertheless, the passive EPR effect-based accumulation of nanoparticles in the tumor tissues is strongly heterogeneous between different types of cancer and experimental models, and hence active receptor engagement plays a more significant role in increasing cellular specificity in the tumor microenvironment.

The chemistry of sterols may also contribute to the tumor targeting properties of nanoparticles. Replacement of standard cholesterol with its natural analogues allowed for developing ginsenoside Rg3-containing LNPs capable of interaction with overexpressed proteins on tumor cells like GLUT1. These LNPs enabled marking tumor cell antigens and, at the same time, influencing the tumor microenvironment and the immune response to it [65]. Thus, LNP components can play several roles at once to help in the formation of nanoparticles as well as to provide receptor binding or immune modulation.

### 4.4. Brain/CNS Targeting Strategies

Among the organs and tissues hardest to target using lipid-based nanoparticles are those of the central nervous system (CNS). This is because the blood–brain barrier (BBB) is restrictive to most macromolecules and nanoparticles traveling through circulation. Therefore, successful CNS targeting requires the design of methods that enable bypassing the BBB or exploiting the natural transport systems at the brain’s endothelial cells [66].

Among the route-based approaches to bypassing systemic barriers is intranasal delivery [67]. Here, lipid-based nanoparticles together with stimuli-responsive or mucoadhesive formulations increase their residence in the nasal cavity, facilitating their transport to the brain through olfactory or trigeminal pathways. While this method has potential to minimize systemic exposure and accumulation in the liver, it strongly depends on nanoparticle properties and mucociliary clearance [67].

In the case of systemic delivery, receptor-mediated transcytosis provides another possible approach. Functionalization of lipid-based nanoparticles with transferrin or lactoferrin and other BBB receptors allows for enhancing their uptake by the brain endothelium and their transportation into CNS tissue. The idea here is not to alter BBB integrity but to utilize the endogenous transport system [68]. Notably, the uptake of nanoparticles by brain endothelial cells alone is not considered to be sufficient proof of nanoparticle transcytosis across the blood–brain barrier; thus, they have to be measured separately.

Among the latest developments in LNP design are computational screenings combined with cell-specific targeting [69]. LNPs containing mRNA that is functionalized with acetylcholine through acetylcholine-conjugated DMG-PEG2000 demonstrated about 3.6-fold higher brain mRNA expression compared with non-targeted LNPs. The targeted LNP preferentially delivered mRNA to neuronal and astrocytic cells. Screening was performed using both experimental approaches and graph neural network computational prediction [69]. Altogether, these examples illustrate that CNS delivery is a multistage process including accessing the brain, crossing or bypassing the BBB, and successful delivery to a specific neuron population.

### 4.5. Heart, Kidney, Pancreas, and Other Emerging Targets

Other extrahepatic organs continue to prove challenging due to the absence of the efficient endogenous nanoparticle uptake machinery found in the liver. As a result, current research focuses more on coupling LNP engineering with administration routes, external forces, or even organ-specific endogenous targeting [70].

In particular, cardiac delivery could be greatly affected by the choice of administration route. Intracoronary administration of mRNA-containing LNPs led to widespread mRNA expression in cardiomyocytes, endothelial cells, smooth muscle cells, and fibroblasts in rabbit myocardium. Administration of VEGF mRNA in this way facilitated angiogenesis, decreased fibrosis, and improved cardiac functions after ischemia–reperfusion injury [71]. This case shows how local administration helps circumvent dilution and hepatic sequestration problems without requiring an entirely new LNP composition.

Another possible solution to the problem is physical targeting. A magnetic field could guide the delivery of superparamagnetic iron oxide nanoparticle-loaded LNPs, leading to an increase in cardiac and pulmonary mRNA expression in comparison with unguided delivery [72]. The idea behind this solution is very appealing, as organ targeting could be regulated independently of ligand conjugation to the nanoparticle; however, feasibility will depend on the ability of the magnetic field to penetrate the tissue.

The kidneys present a good example of endogenous protein corona targeting. LNPs containing DOTAP could recruit the circulating vitronectin and form a corona that is able to bind αVβ3 integrins found in clear-cell renal cell carcinoma cells. This kind of endogenous targeting helped increase mRNA expression in kidney tumor models and proved that selective recruitment of the circulating protein could be used for diseased organ targeting without the need to conjugate the ligand to the nanoparticle [73]. This solution resembles the one proposed for ApoE-mediated hepatic targeting; however, the LNP is now targeted towards another protein–receptor pair.

The same techniques could be used to improve the efficiency of pancreatic delivery. Addition of cholecalciferol as the fifth LNP component resulted in a formulation that showed high mRNA expression due to vitamin-D-receptor-mediated targeting and enabled gene editing in pancreatic β cells [74]. Another approach consisted of lipid ratio optimization coupled with intraperitoneal administration of LNPs, which showed much higher expression in pancreatic tumors than in the liver. Delivery of IL-12 mRNA using these particles changed the immune microenvironment of the tumor and improved treatment when used in conjunction with immune checkpoint blockade [75].

Thus, current solutions suggest that there will be no single LNP design enabling effective targeting of extrahepatic organs. Instead, organ-selective delivery could be achieved through protein-corona-dependent LNP composition, endogenous targeting, active ligand targeting, route engineering, biomimicking of the LNP surface, or physical targeting. Organ- and cell-specificity of delivery will depend on the anatomical barriers associated with the target organ and the target cell population within the organ. Therefore, the transition from liver-specific LNPs to programmable delivery will require not only accumulation of LNPs in the organ but also their delivery to the relevant cells inside of each organ. Representative strategies for extrahepatic and organ-selective LNP delivery are summarized in Table 2.

## 5. Active and Endogenous Targeting Strategies

Cellular specificity in a target organ outside of the liver demands further regulation regarding how LNPs are recognized by different cell types. In general, there are two different approaches that could accomplish the aforementioned goal. Active targeting refers to modification of the surface of nanoparticles using exogenous ligands to interact with cell surface receptors, whereas endogenous targeting makes use of proteins and other molecules present in the biological environment to form a targeting interface. The above-mentioned approaches are becoming increasingly intertwined, as engineered ligands and lipids, as well as the protein corona, are used to achieve both goals at the same time.

### 5.1. Antibodies, Peptides, Aptamers, and Small Molecules

Functionalization of LNP surfaces with targeting ligands is an obvious approach to improve the affinity of nanoparticles towards certain cell populations. Antibodies, peptides, aptamers, carbohydrates, or small molecules can be coupled to lipid or PEG moieties at the surface of LNPs. Thus, a passively targeting carrier becomes an actively targeting delivery vehicle by becoming recognized by a certain receptor. The efficiency of this targeting approach, however, relies not only on the selection of ligands but also on their density, orientation, linker structure, and availability over the surrounding PEG layer.

Antibodies and antibody fragments provide highly specific binding to cell surface proteins and have been extensively applied to tumor targeting and immunotherapy. Nanocarriers with surface-engineered antibodies or fragments to HER2 and EGFR receptors promote multivalency and receptor-mediated uptake of these particles [76,77]. On the other hand, peptides can provide a smaller alternative to antibodies while still retaining their binding ability. Folate and its analogs were used to target overexpression of certain receptors by tumors. PEG linkers are usually introduced to increase the availability of ligands over the surface of nanoparticles in order to avoid any steric barriers provided by lipids [76,77].

The diversity of potential ligands is an essential advantage when dealing with organs or tissues with multiple specialized cell populations. For example, for the brain, antibodies targeted to vascular or immune cell surface proteins, peptide ligands such as RVG-29 and Angiopep-2, DNA aptamers, or choline-containing small molecules can be considered for transport across the blood–brain barrier and following recognition by neurons, astrocytes, microglia, and other cell populations of the nervous system [78]. The main advantage of these approaches is that tissue access and cell recognition can be achieved through separate stages of the process.

Small carbohydrate-derived ligands also serve as examples of well-defined receptor binding. Phenyl β-D-galactoside derivatives coupled to lipid/calcium/phosphate nanoparticles improved recognition of the asialoglycoprotein receptor (ASGPR), leading to more efficient delivery of VEGF siRNA to hepatocellular carcinoma cells. In the orthotopic tumor model, such an approach decreased VEGF expression, tumor vascularization, primary tumor growth, and distal metastasis [79]. Overall, different ligand families can improve recognition by certain cells, but their overall efficiency relies on the efficient connection of receptor recognition with delivery into cells [70].

### 5.2. Receptor-Mediated Targeting

The core of active targeting does not solely rely on the increase in nanoparticle adhesion to target cells, as it involves engaging receptors that could support efficient internalization. The expression level, tissue distribution, recycling, and intracellular trafficking pathway of receptors are thus important parameters for evaluating targeting efficiency [80].

The galactose ASGPR interaction has served as a prototype of the targeted nanoparticle–receptor interaction. The presence of multivalently displayed galactose residues in functionalized LNPs would allow for taking advantage of high ASGPR expression in hepatocytes and improve receptor affinity due to the cluster glycoside effect. Post receptor interaction, nanoparticles are internalized in a clathrin-dependent and calcium-triggered manner and proceed with their endosomal trafficking. This example demonstrates how the density of ligands and the biology of receptors may be utilized for increasing cell-specific delivery efficiency [81].

It has also been shown that multicomponent LNP compositions could be successfully used for improving targeting. Modification of MC3/DSPC/cholesterol/DMG-PEG2000 nanoparticle composition with Gal-PEG2000-DSPE has led to 3.5-fold increase in uptake and 3.9-fold accumulation of nanoparticles in hepatocytes through ASGPR-based targeting. The competitive inhibition of receptor activity was able to decrease nanoparticle internalization, indicating that improved uptake was mediated by ligand–receptor interaction and not just nonspecific accumulation [82].

However, it is important to note that increased receptor-mediated internalization does not necessarily mean successful delivery of nucleic acids. In addition to receptor binding and internalization, LNPs should also escape from the endosomes, release their cargo, and deliver it to the appropriate intracellular machinery. Thus, an efficient targeting receptor should provide both selective binding and appropriate intracellular trafficking pathways. This is especially important when taking into account the effect of the protein corona.

### 5.3. Combining Ligand Targeting with Corona Engineering

This distinction is becoming increasingly blurred as well. A synthetic ligand does not function in isolation; upon systemic administration, ligand-functionalized LNPs face a pool of plasma proteins that can mask, modify, and complement interactions of ligands with their corresponding receptors. On the other hand, protein corona engineering can produce receptor targeting without using a synthetic ligand at all. Therefore, the future generation of LNP-targeting technologies will likely require simultaneous engineering of both interfaces.

Engineering of the protein corona alone is enough to create cell-specific receptor interactions. The formulation of a multicomponent LNP composed of DOTAP, DC-cholesterol, cholesterol, DOPE, and DOPE-PEG2000 spontaneously developed a protein corona rich in APOA1, haptoglobin, and albumin. The developed biological identity of the LNP was associated with increased recognition of the receptor MARCO on Kupffer cells and allowed for successful siRNA-mediated downregulation of TIMP1 in hepatic macrophages. As a result of the downregulation of matrix metalloproteinase activity, liver fibrosis development was attenuated in the murine model [83]. This example clearly shows how a rational choice of lipid formulation can result in the creation of the receptor targeting interface without the use of a synthetic ligand conjugated on the surface.

Combining detargeting from the liver with active retargeting to extrahepatic cells is another efficient strategy. The use of engineered receptor-binding-defective variants of ApoE or manipulation of the PCSK9–LDLR axis decreases the uptake of the nanoparticle via interaction with LDLR in the liver, while conjugation of antibodies allows for targeting via different receptors [84]. These two approaches provided a reduction in off-target liver transduction up to 90% while retaining receptor-specific delivery of the LNP to targeted extrahepatic cells. At the same time, retargeting by means of antibodies remained efficient in the presence of a corona with the engineered detargeting interface, which indicates the possibility of combining these two strategies instead of competing with them [84].

Another way to combine both synthetic and endogenous receptors is the use of peptides. Display of short peptide sequences or modular lipopeptides on the LNP surface can affect nano–bio interactions and modify the subsequent formation of a protein corona. In this case, a peptide sequence does not function only as a receptor ligand but rather modifies the biological identity of the LNP and thus affects its organ distribution and cellular recognition [85]. This approach expands active targeting from the simple ligand receptor scheme to a complex process where the synthetic surface chemistry programs subsequent biological interactions.

Thus, it becomes clear that precise LNP targeting requires simultaneous management of synthetic and biological interfaces. Antibodies, peptides, aptamers, carbohydrates, and small molecules can provide receptor specificity, while lipid composition and surface chemistry determine which proteins will form the biological interface in the further course. Combination of hepatic detargeting, controlled protein recruitment, and receptor-specific retargeting can provide more precise cellular targeting than each of them separately. Nevertheless, ultimately, the success of targeting has to be determined by the functional delivery of nucleic acids into the cytoplasm, rather than the mere accumulation or cellular uptake of the nanoparticle, as receptor engagement and internalization do not guarantee delivery if the cargo is sequestered or degraded within endolysosomal compartments. Key active and endogenous strategies for cell-selective LNP targeting are summarized in Table 3.

## 6. Challenges and Emerging Technologies

Although significant strides have been made toward steering LNPs away from hepatic tropism, making them reproducibly cell-specific and biologically active continues to prove difficult. Biodistribution at the organ level merely represents the first step in a multi-step delivery process involving not only targeting of the correct cell population but also entry into those cells, escape from endosomes, release into the cytosol, and functionality of the nucleic acid cargo. In addition, formulations that work well under idealized conditions might behave differently in different animal models or even humans, and immunogenicity and toxicity become ever more important issues when repeat dosing is required. Major challenges and emerging strategies for programmable extrahepatic LNP delivery are summarized in Table 4.

### 6.1. Organ Targeting Versus Cell-Specific Functional Delivery

The ability to distinguish physical nanoparticle accumulation and functional nucleic acid delivery is one of the key challenges in the development of LNPs. Determination of total LNP or cargo amounts in an organ provides information about pharmacokinetics of delivery but cannot guarantee the fact that the therapeutic cargo was delivered successfully to its target cell population, left the intracellular vesicles, and had the desired effect on the biology of the cells [95].

Functional screening assays showed the difference between these parameters. Co-delivery of molecular barcodes and Cre mRNA into the reporter animals allowed for identifying the cells in which Cre recombination was successfully performed. Functional screening techniques proved that nanoparticle presence in an organ does not always mean that gene delivery and its expression took place in individual cell populations [87]. Therefore, biodistribution has to be more carefully studied at the cellular level rather than at organ levels only.

The importance of functional delivery is exemplified by organ-specific protein expression and activity. Delivery of mRNA coding bispecific T-cell engager against glypican-3 via LNP led to the creation of therapeutic protein only in the hepatocellular carcinoma tissue with decreased concentration in systemic circulation and, consequently, decreased toxicity [96]. Thus, one of the major advantages of the delivery of mRNAs can be used as the targeted tissue will act as a bioreactor producing therapeutic protein locally without delivering it systemically.

Intracellular delivery of the cargo is also crucial after the LNP enters the target organ. Systematic study of the effect of phospholipids on the SORT LNP showed that reduction in helper phospholipid can lead to loss of functionality of nanoparticles despite their successful formation and delivery. Nanoparticles became unable to deliver mRNAs in the cytosol as their membrane fusion and endosomal escape was hindered [88]. This shows one of the key concepts in developing LNPs that target organs outside of the liver: tropism to an organ, nanoparticle uptake by the cells, and cytosolic delivery are connected but separate parameters.

### 6.2. Endosomal Escape and Intracellular Delivery

LNP particles must destabilize the endosomal membrane enough to deliver the drug out of the endosome before it is routed for lysosomal degradation. However, the endosomal membrane must be damaged in a controlled manner, as otherwise there might be inflammation and cytotoxic effects associated with such excessive damage of the membranes [89].

Recent research shows that efficient escape can be achieved without damaging the endosomal membrane excessively. LNPs made of biodegradable multi-tailed ionizable lipids, such as 4A3-SC8, cause relatively small amounts of membrane damage and still ensure efficient mRNA delivery. In doing so, they limit membrane damage to small openings, which allows ESCRT repair and prevents triggering of galectin-dependent inflammation pathways. Furthermore, pharmacological blockage of galectin-dependent membrane damage sensing mechanisms reduces inflammation without compromising delivery efficiency [89]. This suggests that in addition to maximizing endosomal membrane damage, LNP composition should allow for transient opening sufficient for cargo release.

New technology developments also contribute to better quantification of this process. The FLINT method uses redox-sensitive FRET-FLIM labeling of mRNA, which allows for distinguishing between internalization of the cargo and its release to the cytosol and damage of the membrane [90]. This approach provides spatial and time-resolved data about the process, which goes beyond conventional bulk uptake measurements and helps to identify the formulation where cellular internalization is accompanied by efficient delivery to the cytosol.

Other inspiration could come from biological vesicles. Extracellular vesicles use several strategies, including membrane fusion, pore formation, and membrane remodeling, to enable cytosolic release of cargo [97]. This inspired the development of biomimetic LNP design, including cone-shaped ionizable lipids, a fusogenic membrane, and pH-responsive components [97]. While the efficiency of endosomal escape differs depending on the experimental setup used, improving this process is vital, as even highly specific organ and cell targeting will be insufficient if cargo will remain inside of the endolysosomes.

### 6.3. High-Throughput and Barcoded LNP Screening

The vast chemical and formulation space available for LNP development makes one-formulation-at-a-time testing of candidates less and less efficient. Each of a number of parameters such as the ionizable lipid structure, the helper lipid identity, cholesterol chemistry, the PEG lipid structure, molar ratios, nanoparticle characteristics, and drug characteristics may impact biological performance. Thus, high-throughput screening becomes an ever more promising approach for developing organ- and cell-selective LNPs [98,99].

Multiparametric in vitro tests may improve the selection of candidate LNPs. A high-throughput system that evaluates the blood–brain barrier tested both endothelial transfection and nanoparticle penetration across brain endothelial monolayer, thereby discovering LNP formulations with the improved potential for central nervous system delivery. Importantly, endothelial transfection and crossing of the barrier were not correlated, demonstrating that in general each step of the delivery process needs to be tested separately and that the simple potency test in vitro is not necessarily predictive of in vivo behavior [100].

Barcoding technology allows for screening much larger LNP libraries in vivo. The direct integration of the sequence barcodes into the mRNA allows for the tracking of individual LNPs following pooled injection while keeping the physicochemical connection between the delivery vector and cargo. Testing 122 LNP formulations in wild-type and ApoE-deficient mice discovered extrahepatic formulations, including the spleen-targeting LNP formulation capable of transfecting antigen-presenting cells and producing a therapeutic effect in the melanoma model [99]. Such approaches can greatly speed up the discovery of formulations that would otherwise be hard to identify.

Of note, future screening platforms have to focus on functional endpoints instead of LNP accumulation per se. Combining barcoded delivery with single-cell analysis, reporter assays, and cytosolic activity tests will allow for differentiating between LNPs that are just delivered to the organ and those delivering the therapeutic agent to the target cells.

### 6.4. Species Differences and Clinical Translation

One of the key translational hurdles is the unreliability of LNP behavior in cultured cells or animal models as an indicator of human efficacy. Protein corona composition, receptor expression, immunological reactions, tissue structure, metabolic processes, and vascular physiology might all differ in experimental settings and different species and affect both the biodistribution and intracellular delivery of nanoparticles.

But even before interspecies comparison becomes an issue, there is a gap that might appear between preclinical tests and experiments. The comparative investigation of LNPs with clinically relevant ionizable lipids revealed that the in vitro mRNA expression level does not predict in vivo protein expression levels, and differences in in vivo protein expression do not always correlate proportionally with the vaccine-induced antibody response [91]. Thus, seemingly identical physicochemical characteristics can produce different biological effects depending on the endpoint under study.

There is also the issue of a disease-specific microenvironment. ROS-responsive LNPs encapsulated in a temperature-sensitive hydrogel allowed for localized delivery of IL-4 mRNA in the case of diabetes and simultaneously influenced the local oxidative environment [101]. While these therapeutic results of LNP application demonstrated in rodents represent the ability of microenvironment-responsive delivery, they still need to be confirmed in experimental conditions replicating the human tissue structure and the pathology itself.

More generally, research in nanomedicine suggests that significant differences between rodents and humans due to anatomy and metabolic features can cause large differences in therapeutic effects [102]. Thus, in addition to finding efficient LNPs and performing appropriate preclinical tests, the development of relevant tissue models of human organs is required to bridge this gap.

### 6.5. Safety and Repeated Dosing

Safety standards for LNPs become stricter for nucleic acid therapeutics requiring either chronic or repeated administration. The possible drawbacks of such treatments include inflammation due to ionizable lipids, complement activation, development of anti-PEG antibodies, accelerated blood clearance, and tissue toxicity. Formulation development requires optimization of delivery potency in accordance with biodegradability and immunogenic safety [93].

The effect of PEG lipids becomes especially crucial in the case of repeated drug administration. LNPs containing shortened DMG-PEG2000 in lower molar concentrations revealed higher rates of PEG shedding, low levels of anti-PEG IgM production, and complement activation, and mRNA expression was preserved after repeated intravenous administration [93]. These data suggest that shedding kinetics of the PEG anchor could affect the accelerated blood clearance effect and should be considered when designing chronic formulations.

Another direction for increasing the safety of LNPs includes modification of the ionizable lipid chemistry. LNPs using the ionizable glutarimide-containing lipid MOP-1 revealed good cell viability and safety profiles despite high-dose experimental conditions while maintaining high vaccine immunogenicity [94]. Metabolically labile ionizable lipid molecules serve as an example of how biodegradability could decrease lipid-induced toxicity without loss of delivery efficiency.

Testing in large animals also plays a crucial role before clinical use. In pigs, siRNA LNPs targeted to fibrinogen showed a high level of dose-dependent suppression without any complement-activation-related pseudo-allergy and absence of major toxicity on liver, kidney, and hematologic functions under described conditions. Of note, the biological consequence of high levels of fibrinogen silencing could be reversed by exogenous fibrinogen administration [92].

### 6.6. AI-Guided LNP Design

With the advent of LNP designs towards multidimensional engineering, artificial intelligence and machine learning techniques serve as an additional tool to screen potential lipid formulations via high-throughput experiments [3,99,103]. While experimental screening allows for empirical evaluation of each possible lipid and formula, computational methods can decipher relationships between molecule, particle properties, formulation composition, and biological activity and further select the most promising candidates for validation by experiments [3,99,103].

Machine-learning-based frameworks combined with optimization algorithms can examine such features as particle size, surface charge, PEG content, and the presence of targeting ligands to determine formulation profiles capable of achieving an optimal ratio of efficiency and tolerability [103,104]. However, the applicability of such methods strongly depends on the quality and biological relevance of the input data used for building and training the models [103,104].

Generation and prediction can be applied to discover ionizable lipids as well. Machine learning techniques can rank or generate the most promising candidate lipid formulas, thus reducing the number of molecules needed for synthesis and high-throughput screening. In more advanced architectures, it is possible to incorporate 3D representations of the molecules along with the formulation ratios and process parameters, making it possible to consider both the chemical nature of the lipid and the formulation in order to predict transfection performance [105,106].

The role of AI in the design of liposomes should be considered as an additional element to the experiment rather than as a replacement of the experiment and in vivo screening. Efficient drug discovery pipelines can involve such steps as computational candidate generation, automated formulation, high throughput physicochemical characterization, barcoding in vivo screening, single-cell analysis, and repeated re-training of the model [3,99,103].

All things considered, all of the factors above indicate that next-generation LNPs cannot rely only on maximizing delivery efficiency. Successful extracellular platforms require simultaneous optimization of organ distribution, cell specificity, intracellular transport, endosomal escape, toxicity, and stability. Combining biological mechanisms with high-throughput screening and computational methods can lead to the development of LNPs that can go beyond empirical optimization and become predictable and programmable [99].

### 6.7. Manufacturing and Scalability Aspects

Increasingly complex organ- and cell-targeted lipid nanoparticle (LNP) formulations pose some significant manufacturing issues. Scale-up of formulations from lab scale to clinical grade production involves the reproducible control of important quality attributes such as particle size and polydispersity, lipid mixture composition, nucleic acid encapsulation efficiency, surface characteristics, activity, and stability [6]. The controlled mixing approach and microfluidics technology could assist in the development of scalable and reproducible LNP manufacture [44]; nevertheless, adding additional lipid components, targeting molecules, surface coupling, or biomimicking coating might complicate the process even more, imposing the need for more purification and quality control steps [33].

## 7. Conclusions and Future Perspectives

### 7.1. Emerging Principles for Escaping ApoE-Driven Liver Tropism

The traditional view that systemic LNP particles have inherent liver tropism is now giving way to a more dynamic paradigm where biodistribution emerges from the complex interplay of nanoparticle composition and biology. One key example is ApoE-mediated liver targeting, whereby nanoparticle lipid composition and surface properties determine ApoE binding that may lead to subsequent receptor-mediated liver targeting. Importantly, the examples outlined here illustrate that this mechanism is neither ubiquitous nor inevitable. Ionizable lipid composition, lipid compositions, sterols, PEG-lipids, surface charge, morphology and even mode of administration all have an impact on the biological identity and biodistribution of LNPs [4].

The most compelling example of this phenomenon is sterol engineering. Replacement of conventional cholesterol with endogenous corticosteroid-derived lipids, corticosterone and progesterone, significantly decreases the delivery of particles to the liver and leads to almost 100% selective mRNA expression in the spleen [43,107]. This is associated with a change in the protein corona towards enrichment in β2-glycoprotein I, suggesting that avoidance of ApoE-mediated liver tropism involves redirection of targeting to another endogenous protein receptor [43,107]. Similarly, SORT strategies show that changes in surface charge, lipid chemistry, or sterol modification can affect the formation of the protein corona and redirect the delivery of functional RNA into the lungs and spleen, rather than the liver [33]. Overall, it can be concluded that the formation of the protein corona is not a simple hurdle to delivery but an engineerable interface enabling organ-specific targeting.

### 7.2. Toward Programmable Organ- and Cell-Selective LNPs

Next, the task is that of moving beyond organ-specific accumulation towards reproducible, cell-specific functional delivery. Modern LNP development is increasingly modular in nature: the chemistry of lipids can define organ tropism, recruitment or targeting ligands can target specific cells, and intracellularly responsive systems can control release and function of payloads [33,108]. This multilevel engineering will be especially important for genome editing drugs that may result in irreversible side effects when expressed in non-target cells.

Targeting of active ligands adds another layer of cell specificity. The targeting of abnormal surface receptors by means of VCAM-1-targeted lipid nanocarriers for diseased lung myofibroblasts illustrates a way to select cell populations for therapeutic use in a complex organ environment [109]. Nevertheless, next-generation LNPs should address issues of efficient delivery, targeting with the ability to escape from endosomes, biodegradability, safety with repeated administrations, and high efficiency across species.

In the end, targeted LNP delivery will require a transition from optimization of individual formulation parameters to integration of lipid chemistry, formation of protein corona, interaction with receptors, cellular traffic, and functional activity of payloads. The combination of mechanistic engineering with barcoded screening, functional analysis at the single cell level, human-relevant modeling and computation may allow LNPs to move beyond the realm of liver-targeting carriers and become organ and cell-specific nucleic acid therapeutics. While there have been significant developments in the engineering of pre-clinical LNP systems, the translation of LNP technology to the clinic has largely been confined to a relatively smaller number of delivery scenarios. Approved LNP-based nucleic acid drugs include siRNA delivery to the liver and mRNA vaccination through the intramuscular route, whereas several LNP systems have been developed for other disease conditions and are currently being evaluated in clinical trials. This is illustrated in Table 5.

## Figures and Tables

**Table 1:** Key engineering variables influencing LNP tropism and ApoE-dependent liver targeting.

Engineering Variable	Representative Modification	Effect on Tropism	Administration Route	Reference
Ionizable lipid structure	cKK-E12 → cKK-E15	Reduced ApoE/LDLR dependence	Intravenous	[26]
Ionizable lipid chemistry	5A2-SC8 vs. 3A5-SC14	Shifted hepatocyte/Kupffer-cell tropism	Intravenous	[37]
Ionizable lipid/pKa	Oxabispidine lipid	Enhanced lung expression	Intratracheal/inhalation	[35]
Ionizable phospholipid structure	Multi-tailed iPhos	Organ-selective mRNA/CRISPR delivery	Intravenous	[9]
Ionizable lipid headgroup	Piperazine-containing lipids	Enhanced immune cell mRNA delivery	Intravenous	[9]
Biodegradable ionizable lipid	A^3^-lipid structural evolution	Improved activity and biodegradability	Intravenous/intramuscular	[39]
ML-guided ionizable lipid	Combinatorial lipid library	Identification of potent muscle/immune cell delivery	Intravenous/intramuscular	[3]
Helper lipid	Monoolein replacing DOPE	Enhanced lung/spleen expression	Intranasal (lung); intravenous (spleen)	[40]
Sterol chemistry/morphology	Modified cholesterol/sterol	Reduced ApoE and liver tropism	Intratumoral	[29]
PEG lipid chemistry	PEG density/anchor length	Altered protein binding and distribution	Intravenous	[23,47]
Surface charge/SORT lipid	Charged SORT lipid	Redirected delivery to lung/spleen	Intravenous	[5,12]
Surface modification	Polyanion coating	Reduced protein adsorption; altered distribution	Intravenous	[27]

Abbreviations: ApoE, apolipoprotein E; DOPE, 1,2-dioleoyl-sn-glycero-3-phosphoethanolamine; LDLR, low-density lipoprotein receptor; LNP, lipid nanoparticle; MC3, DLin-MC3-DMA; PEG, polyethylene glycol; pKa, acid dissociation constant; SORT, selective organ targeting; ML, machine learning.

**Table 2:** Representative LNP strategies for extrahepatic and organ-selective delivery.

Target Organ	LNP Engineering Strategy	Key Targeting Outcome	Administration Route	Reference
Lung	Cationic helper lipid (18:0 DDAB)	Enhanced pulmonary endothelial cell delivery	Intravenous	[55]
	Asymmetric sulfonium ionizable lipids	Lung-selective mRNA expression and reduced inflammation	Intravenous	[56]
	Oxabispidine-based ionizable lipid	Enhanced pulmonary mRNA expression	Intratracheally	[35]
Spleen	Zn(II)-coordinated imidazole LNPs	Enhanced splenic mRNA delivery and T-cell responses	Intramuscular	[58]
	TOT-5 lipid with increased DSPC	Complement-mediated splenic B-cell targeting	Intravenous	[59]
Tumor	Ginsenoside Rg3-containing LNPs	Enhanced tumor cell interactions and immune modulation	Intravenous	[65]
Brain/CNS	Acetylcholine-functionalized LNPs	Enhanced neuronal and astrocytic mRNA delivery	Intravenous	[69]
Heart	Intracoronary VEGF mRNA-LNPs	Improved angiogenesis and cardiac function	Intracoronary	[71]
Kidney tumor	DOTAP-containing LNPs	Vitronectin-mediated renal tumor targeting	Intravenous (tail vein)	[73]
Pancreas	Cholecalciferol-modified LNPs	Enhanced pancreatic β-cell gene editing	Intravenous	[74]
Pancreatic tumor	Optimized LNPs with intraperitoneal delivery	Increased tumor mRNA expression and antitumor activity	Intraperitoneal	[75]

Abbreviations: CNS, central nervous system; DDAB, dimethyldioctadecylammonium bromide; DOTAP, 1,2-dioleoyl-3-trimethylammonium-propane; DSPC, 1,2-distearoyl-sn-glycero-3-phosphocholine; LNP, lipid nanoparticle; mRNA, messenger RNA; VEGF, vascular endothelial growth factor; Zn(II).

**Table 3:** Active and endogenous targeting strategies for cell-selective LNP delivery.

Targeting Strategy	Targeting Ligand/Mechanism	Key Advantage	Reference
Antibody targeting	Antibody-mediated receptor recognition	High cell specificity	[76,86]
Peptide targeting	Targeting peptides and receptor interactions	Small, tunable ligands	[78]
Aptamer targeting	DNA/RNA aptamer–receptor binding	Programmable cell targeting	[36,78]
Small-molecule targeting	Folate-mediated receptor targeting	Enhanced tumor cell delivery	[76,77]
Carbohydrate targeting	GalNAc–ASGPR recognition	Selective hepatocyte delivery	[79,82]
ApoE-mediated targeting	ApoE–LDLR interactions	Endogenous hepatic targeting	[4,30]
SORT-mediated targeting	Formulation-dependent protein corona	Lung/spleen targeting	[5]
Complement-mediated targeting	Complement C3–CD21/35 interactions	Splenic B-cell targeting	[59]

Abbreviations: ApoE, apolipoprotein E; ASGPR, asialoglycoprotein receptor; C3, complement component 3; CD21/35, complement receptors 2 and 1; CNS, central nervous system; LDLR, low-density lipoprotein receptor; LNP, lipid nanoparticle; PEG, polyethylene glycol; SORT, selective organ targeting; αVβ3, integrin alpha V beta 3.

**Table 4:** Major challenges and emerging solutions for extrahepatic LNP delivery.

Challenge	Key Issue	Emerging Solution	Reference
Functional delivery	Organ accumulation ≠ functional cargo delivery	Functional reporters and single-cell analysis	[87]
Endosomal escape	Cargo may remain trapped in endosomes	Optimize fusogenic/helper lipid chemistry	[88]
Endosomal damage	Excess membrane disruption can cause inflammation	Biodegradable lipids with controlled membrane disruption	[89]
Escape measurement	Uptake does not indicate cytosolic release	FRET-FLIM-based escape assays	[90]
Species translation	LNP performance may differ across species	Human-relevant and large animal models	[91,92]
Repeated dosing	Anti-PEG immunity and accelerated clearance	Optimize PEG content and shedding kinetics	[93]
Lipid toxicity	Potency may conflict with tolerability	Biodegradable/metabolically labile ionizable lipids	[89,94]

Abbreviations: DMG-PEG2000, 1,2-dimyristoyl-rac-glycero-3-methoxypolyethylene glycol-2000; FLINT, fluorescence lifetime imaging of nucleic-acid trafficking; FRET-FLIM, Förster resonance energy transfer–fluorescence lifetime imaging microscopy; LNP, lipid nanoparticle; mRNA, messenger RNA; PEG, polyethylene glycol.

**Table 5:** Representative approved and clinically validated LNP-based nucleic acid therapeutics.

Product/Candidate	Cargo	Indication	Target Organ/Tissue	Administration Route	Clinical Status	Reference
Patisiran (ONPATTRO)	siRNA targeting TTR mRNA	Hereditary transthyretin-mediated amyloidosis with polyneuropathy	Liver/hepatocytes	Intravenous	FDA approved	[110]
COMIRNATY (BNT162b2)	Nucleoside-modified mRNA encoding SARS-CoV-2 spike antigen	COVID-19 prevention	Muscle/immune system	Intramuscular	FDA approved	[111]
SPIKEVAX (mRNA-1273)	Nucleoside-modified mRNA encoding SARS-CoV-2 spike antigen	COVID-19 prevention	Muscle/immune system	Intramuscular	FDA approved	[112]
Intismeran autogene (V940/mRNA-4157)	Personalized neoantigen-encoding mRNA	Solid tumors, including melanoma and NSCLC	Muscle/immune system	Intramuscular	Phase 3	[113]
ITI-5000	Self-amplifying RNA encoding tumor-associated antigens	Triple-negative breast cancer	Muscle/immune system	Intramuscular	Phase 1	[114]

Abbreviations: COVID-19, coronavirus disease 2019; FDA, U.S. Food and Drug Administration; LNP, lipid nanoparticle; mRNA, messenger RNA; NSCLC, non-small cell lung cancer; saRNA, self-amplifying RNA; siRNA, small interfering RNA; TTR, transthyretin; TNBC, triple-negative breast cancer.

## Data Availability

No new data were created or analyzed in this study. Data sharing is not applicable to this article.
